# Serious disease risk among patients with unexpected weight loss: a matched cohort of over 70 000 primary care presentations

**DOI:** 10.1002/jcsm.13056

**Published:** 2022-09-03

**Authors:** Diana R. Withrow, Jason Oke, Claire Friedemann Smith, Richard Hobbs, Brian D. Nicholson

**Affiliations:** ^1^ Nuffield Department of Primary Care Health Sciences, Medical Sciences Division University of Oxford Oxford UK

**Keywords:** Weight loss, Primary health care, Diagnosis, Differential diagnosis

## Abstract

**Background:**

Unexpected weight loss (UWL) in patients consulting in primary care presents dilemmas for management because of the broad differential diagnoses associated with UWL. Research on the risks of serious disease among patients with UWL to date has largely taken place in secondary care, limiting generalizability to primary care patients. In this study, we use a large matched cohort study to estimate the risks of 12 serious diseases among patients presenting to primary care with UWL where this was recorded, stratified by age and sex, in order to inform a rational clinical approach to patients presenting with UWL.

**Methods:**

This was a retrospective matched cohort study using electronic health records (EHRs) from the UK Clinical Practice Research Datalink (CPRD). Each patient with UWL (ascertained from EHR coding) was matched to five patients without UWL and followed until the earliest of a diagnosis of the serious disease, date of death, exit from the CPRD database, or end of the study. Observed absolute risks of the 12 serious diseases were estimated as probabilities, and hazard ratios (HRs) were estimated with Cox proportional hazards models.

**Results:**

Between 2000 and 2012, 70 193 patients in CPRD had at least one record of UWL and were matched with 295 579 patients without UWL. Patients with UWL had significantly higher risk of nearly all serious diseases examined compared with patients without. HRs ranged from 1.43 for congestive heart failure [95% confidence interval (CI): 1.27–1.62] to 9.70 for malabsorption (95% CI: 6.81–13.82). The absolute risks of any given serious disease were relatively low (<6% after 1 year). The magnitude and rank order of absolute risks varied by age and sex. Depression was the most common diagnosis among women aged <80 with UWL (3.74% of women aged <60 and 2.46% of women aged 60–79), whereas diabetes was the most common in men <60 with UWL (2.96%) and cancer was the most common in men aged 60 and over with UWL (3.79% of men aged 60–70 and 5.28% of men aged ≥80).

**Conclusions:**

This analysis provides new evidence to patients and clinicians about the risks of serious disease among patients presenting with UWL in primary care. Depending on age and sex, the results suggest that workup for UWL should include screening for diabetes, thyroid dysfunction, depression, and dementia. If performed in a timely manner, this workup could be used to triage patients eligible for cancer pathway referral.

## Introduction

Unexpected weight loss (UWL) in patients consulting in the primary care setting presents a diagnostic dilemma for the clinician.^1^ Determining who should be investigated further and who can be spared unnecessary investigation relies on an understanding of the broad differential diagnoses associated with UWL. The possibility of malignancy suggests that the patient may need an extensive workup, but the low probability of malignancy means that unnecessary invasive testing could be conducted, with non‐negligible costs to the healthcare system and the patient.^1^, ^2^ Studies have identified the coexisting symptoms, signs, and blood test abnormalities that increase the probability of an underlying but as yet undiagnosed malignancy in patients with UWL consulting primary care.^3^, ^4^ The wider differential of serious disease diagnoses that also warrant investigation, and how the risk of these diseases compare with risk of cancer, remains poorly defined. Understanding this differential would inform a rational clinical approach to patients presenting with UWL.

Recent reviews have estimated that the probability of malignancy in patients presenting with UWL ranges from 6% to 38%, non‐malignant gastrointestinal disorders from 6% to 19%, cardiopulmonary disorders from 9% to 14%, endocrine disorders from 2% to 11%, and psychiatric disorders from 8% to 33%.^2^, ^5^, ^6^, ^7^, ^8^, ^9^ Many of the studies contributing to these risk estimates have however been based on hospital inpatients or patients referred to secondary care,^10^, ^11^, ^12^, ^13^, ^14^, ^15^, ^16^, ^17^ therefore limiting generalizability to the primary care population where prevalence of serious disease is relatively low. The wide ranges of these published estimates also indicate that even within a secondary care/referred population, there remains broad variation in risk estimates across studies. This is likely due in part to chance as most studies are small (under 200 patients), but the heterogeneity in probabilities reported across studies is also likely to be at least in part due to differences in population demographics and study design; contributing studies have estimated the proportion of patients with each outcome in populations with varying follow‐up time, and age and sex distributions.

In this study, we use a large matched cohort to estimate the absolute and relative risks of a range of diagnoses among patients presenting to primary care with UWL recorded in the medical record, stratified by age and sex. The results could inform clinicians and clinical guidelines on workup for patients presenting with UWL.

## Methods

### Study design and population

**Figure 1:**
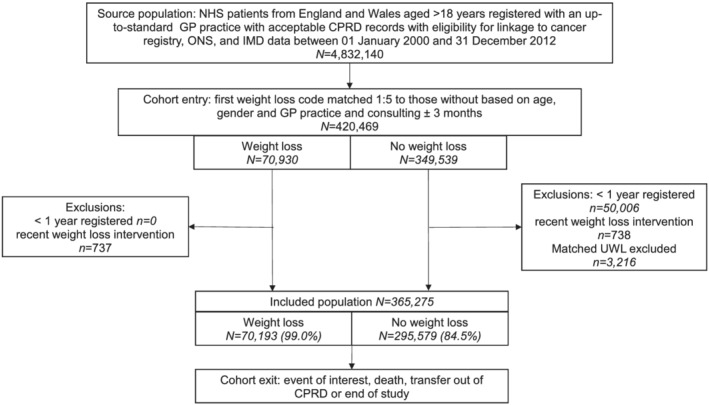
Study population flow diagram. This flow diagram details the source population, cohort entry criteria, exclusion criteria, the sample size of the eligible population with and without unexpected weight loss (UWL), and the study exit criteria. CPRD, Clinical Practice Research Datalink; GP, general practitioner; IMD, index of material deprivation; NHS, National Health Service; ONS, Office of National Statistics.

This was a retrospective matched cohort study using electronic health records from the Clinical Practice Research Datalink (CPRD): a representative anonymized primary care records database covering 6.9% of the UK population.^18^ After acceptance by CPRD's Independent Scientific Advisory Committee (16_164A2A), a protocol was published, which includes this investigation under Aim 2.3.^19^ Patients were eligible for inclusion if they were aged 18 and older and visited a general practitioner (GP) between 1 January 2000 and 31 December 2012. Patients were excluded from the study if they were registered with the GP for less than a year or had been prescribed a weight loss medication or had bariatric surgery in the 6 months prior to the index weight loss event. If a case was excluded, its matched comparators were also excluded.^20^ Cohort entry, exclusions, and exit are summarized in *Figure* 1.

### Unexpected weight loss and matching

Patients were classified as having had UWL if they had at least one Read code for UWL recorded in the electronic health record. Of 120 weight‐related codes, a previous internal validation study identified eight codes, which designated unintentional weight loss and equated to a mean weight loss of 5% or more within a 6 month period. A code of UWL could arise from a range of clinical scenarios, including UWL reported as the patient's presenting condition, after targeted history taking, and after weight measurement as part of the clinical examination or as part of a routine health check or chronic disease review.^21^ Each patient with UWL (case) was matched to five patients without UWL (comparators) with the same year of birth and sex who had consulted in the same practice within 3 months of the patient.

### Subsequent serious diseases

Patients with UWL and their matched comparators were followed up from the date of the index UWL consultation until the earliest of date of diagnosis of the serious disease, date of death, exit from the CPRD database, or end of the study. Our primary analysis included events occurring in the first year following the index UWL consultation. As incident disease was the outcome of interest, only patients without a history of a given disease were included in the analyses for that outcome. Each disease diagnosis was considered independent, and patients were not censored at the diagnosis of another serious disease. Exploratory analyses suggested that on average, fewer than 5% of patients with a given serious disease also had any other serious disease diagnosis in the same year.

The serious diseases included in this analysis were selected based on literature review of diagnoses previously associated with UWL and restricted to those with at least 50 events among cases to permit stratified analyses.^2^ The 12 diseases were alcohol addiction, cancer (any), chronic obstructive pulmonary disorder (COPD), congestive heart failure (CHF), dementia, depression, diabetes, eating disorders, inflammatory bowel disease (IBD), malabsorption, rheumatoid arthritis, and thyroid disorders. Diagnoses were identified using Read codes in CPRD (code lists are available on request from the authors). For cancer, prevalent and incident diagnoses were based on the International Classification of Diseases for Oncology codes in linked National Cancer Registration and Analysis Service cancer registry data.

### Statistical analysis

Observed absolute risks of the 12 serious diseases were estimated as probabilities, stratified and presented by UWL (yes vs. no), age (<60, 60–79, and ≥80), and sex. Numerators were the number of observed events, and denominators were the number of eligible patients in each stratum. Confidence intervals around percentage estimates were estimated using the logit transformation.

Multivariable Cox models were used to estimate hazard ratios (HRs) comparing those with and without UWL. Matching of cases to comparators limited potential confounding by GP, age, and sex. Cox models were performed stratified by match group, whereby coefficients are equal across strata but the baseline hazard is distinct. Smoking status, alcohol consumption, index of material deprivation quintile, number of comorbidities, and body mass index were also included as covariates in the Cox models based on potential associations with UWL and serious disease. The comorbidity count was composed of specific diseases/conditions that could cause weight loss as defined in a previous study.^22^ In the main results, we present HRs for the full first year of follow‐up. To account for differences in the appropriateness of the proportional hazards assumption for different diseases, the HRs for different follow‐up intervals (0–2 and 0–6 months) were also estimated.

## Results

Of just under 5 million eligible National Health Service (NHS) patients registered in CPRD, 70 193 (1.5%) patients had at least one record of UWL and were matched with 295 579 patients without recorded UWL (rUWL) (*Figure* 1). A comparison between patients with and without UWL codes is provided in *Table* 1. Patients with UWL coding were more likely than matched controls to be current smokers (14.6% vs. 8.7%) and to have two or more comorbidities (56.6% vs. 39.0%) but less likely to be overweight or obese (25.1% vs. 51.7%).

**Table 1:** Baseline demographics of the study population by unexpected weight loss status

	Unexpected weight loss		No unexpected weight loss	
	*n*	%	*n*	%
Total	70 193	100	295 579	100
Sex				
Male	29 754	42.4	127 234	43.0
Female	40 439	57.6	168 345	57.0
Mean age (years)	60.0	SD: 20.8	60.4	SD: 20.4
Median age (years)	63.0	IQR: 44–78	64.0	IQR: 45–78
Age group (years)				
18–39	14 394	20.5	56 051	19.0
40–49	8215	11.7	35 093	11.9
50–59	9031	12.9	39 102	13.2
60–69	10 145	14.5	44 617	15.1
70–79	13 550	19.3	59 008	20.0
80+	14 858	21.2	61 704	20.9
Smoking				
Current	10 214	14.6	25 592	8.7
Ex‐smoker	8307	11.8	35 910	12.1
Non‐smoker	15 912	22.7	78 279	26.5
Missing	35 760	50.9	155 798	52.7
Alcohol				
Current	22 588	32.2	98 538	33.3
Non‐drinker	6786	9.7	23 402	7.9
Past drinker	1148	1.6	3133	1.1
Missing	39 671	56.5	170 506	57.7
IMD quintile				
I	13 937	19.9	67 280	22.8
II	14 637	20.9	66 206	22.4
III	14 832	21.1	63 422	21.5
IV	13 352	19.0	52 550	17.8
V	13 381	19.1	46 464	15.7
Comorbidity				
0	13 937	19.9	103 429	35.0
1	16 546	23.6	76 937	26.0
2	14 354	20.4	50 463	17.1
3	10 640	15.2	30 652	10.4
4	6743	9.6	17 094	5.8
5+	7973	11.4	17 000	5.8
BMI group				
Underweight	7560	10.8	5058	1.7
Normal	37 021	52.7	97 974	33.1
Overweight	12 042	17.2	95 206	32.2
Obese	5576	7.9	57 695	19.5
Missing	8001	11.4	40 232	13.6

BMI, body mass index; IMD, index of material deprivation; IQR, inter‐quartile range; SD, standard deviation.

**Figure 2:**
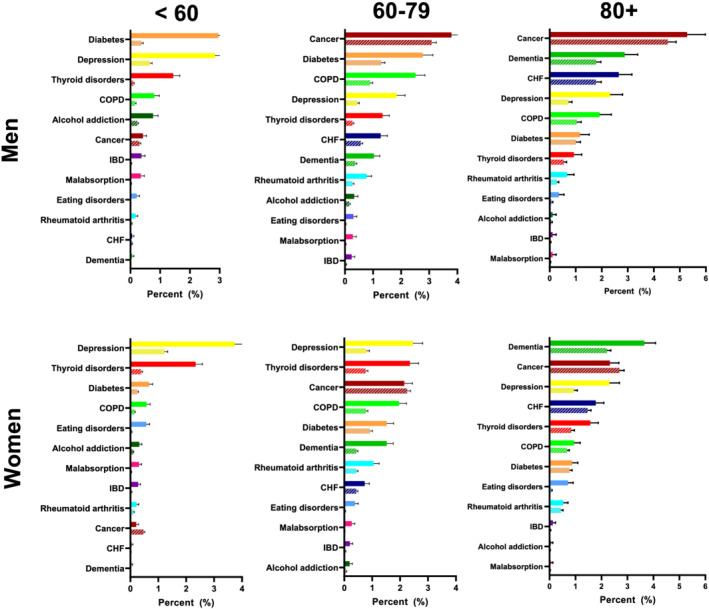
Probability of serious diseases among people with and without unexpected weight loss within 1 year of index unexpected weight loss by age and sex. Solid bars indicate event probabilities among persons with unexplained weight loss. Hashed bars indicate event rates among persons without unexplained weight loss. Error bars indicate 95% confidence intervals around the rates. CHF, congestive heart failure; COPD, chronic obstructive pulmonary disorder; IBD, inflammatory bowel disease.

**Figure 3:**
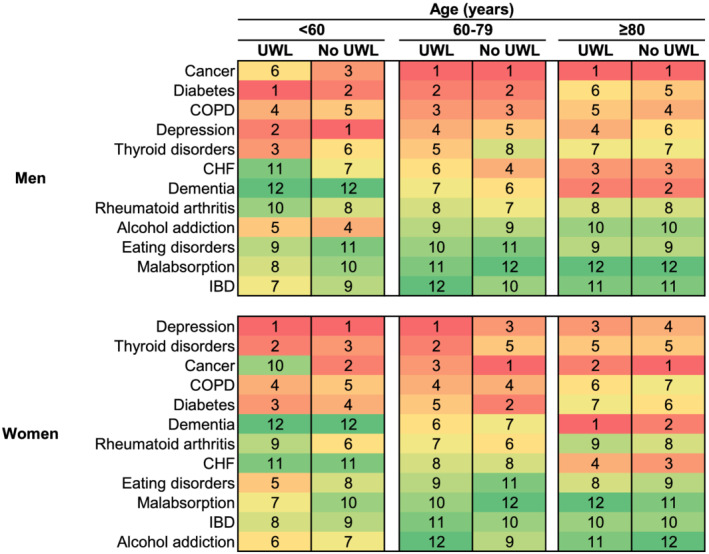
Rank order of diagnoses by observed probability, stratified by age, sex, and unexplained weight loss (UWL). CHF, congestive heart failure; COPD, chronic obstructive pulmonary disorder; IBD, inflammatory bowel disease.

The most common serious disease diagnoses among persons with rUWL were diabetes, cancer, depression, dementia, COPD, and thyroid disorders (*Figures* 2 and 3 and Supporting Information, *Table* S1). The likelihood of these diagnoses varied significantly by age and sex.

### Observed absolute risks: men

Among men aged <60 with rUWL, diabetes was the most common diagnosis [2.96%, 95% confidence interval (CI): 2.67–3.27] followed by depression (2.84%, 95% CI: 2.54–3.18) and thyroid disorders (1.44%, 95% CI: 1.25–1.66, *Figures* 2 and 3 and *Table* S1). Among men aged 60 and older, the three leading diagnoses were the same irrespective of rUWL status, but probabilities of each were higher among men with rUWL than those without. Among men aged 60–79 with rUWL, these diagnoses were in descending order: cancer (3.79%, 95% CI: 3.42–4.19), diabetes (2.77%, 95% CI: 2.45–3.13), and COPD (2.51%, 95% CI: 2.21–2.85). Among men aged 80 and older with rUWL, the three most common diagnoses in descending order were cancer (5.28%, 95% CI: 4.65–5.98), dementia (2.88%, 95% CI: 2.45–3.38), and CHF (2.65%, 95% CI: 2.23–3.16).

### Observed absolute risks: women

Among women aged <80 with rUWL, the most common diagnoses were depression (<60 years: 3.74%, 95% CI: 3.41–4.09; 60–79 years: 2.46%, 95% CI: 2.15–2.81, *Figures* 2 and 3 and *Table* S1) and thyroid disorders (<60 years: 2.34%, 95% CI: 2.12–2.58; 60–79 years: 2.35%, 95% CI: 2.08–2.66). Among women aged <60, the next most common diagnoses were diabetes (0.67%, 95% CI: 0.56–0.80) and COPD (0.58%, 95% CI: 0.48–0.71), whereas among women aged 60–79, the next most common diagnoses were cancer (2.15%, 95% CI: 1.90–2.44) and COPD (1.96%, 95% CI: 1.72–2.23). For women aged over 80 with UWL, the most common diagnoses were dementia (3.65%, 95% CI: 3.27–4.08), cancer (2.32%, 95% CI: 2.02–2.67), depression (2.31%, 95% CI: 1.98–2.69), and CHF (1.78%, 95% CI: 1.52–2.09).

### Hazard ratios

Within a year of the rUWL consultation, patients with rUWL had significantly greater risk of all diagnoses apart from cancer after adjustment for potential confounders (*Table* 2). HRs were highest for some of the least common diagnoses: malabsorption (HR: 9.70, 95% CI: 6.81–13.82), eating disorders (HR: 8.76, 95% CI: 6.71–11.45), and IBD (HR: 7.36, 95% CI: 5.39–10.05). The HRs for thyroid disorders and diabetes were 4.79 (95% CI: 4.34–5.30) and 4.09 (95% CI: 3.69–4.52), respectively. HRs were between 2.00 and 2.99 for depression, alcohol addiction, rheumatoid arthritis, and COPD and between 1.00 and 1.99 for dementia, CHF, and cancer.

**Table 2:** HRs and 95% CIs for risk of serious disease within 12 months among adults with UWL compared with those without

Event		*N*	*n*_events_	HR	95% CI
Malabsorption	No UWL	292 495	71	1	Ref
	UWL	69 706	179	9.70	(6.81–13.82)
Eating disorders	No UWL	290 084	106	1	Ref
	UWL	69 163	295	8.76	(6.71–11.45)
IBD	No UWL	289 079	96	1	Ref
	UWL	69 261	167	7.36	(5.39–10.05)
Thyroid disorders	No UWL	253 354	1081	1	Ref
	UWL	64 112	1148	4.79	(4.34–5.30)
Diabetes	No UWL	246 277	1612	1	Ref
	UWL	63 380	1036	4.09	(3.69–4.52)
Depression	No UWL	182 535	1456	1	Ref
	UWL	51 469	1388	2.92	(2.67–3.19)
Alcohol addiction	No UWL	278 462	297	1	Ref
	UWL	67 410	218	2.61	(2.10–3.23)
Rheumatoid arthritis	No UWL	272 345	627	1	Ref
	UWL	66 922	343	2.49	(2.13–2.91)
COPD	No UWL	260 739	1273	1	Ref
	UWL	64 509	829	2.06	(1.84–2.30)
Dementia	No UWL	280 046	1439	1	Ref
	UWL	67 654	752	1.98	(1.79–2.19)
CHF	No UWL	273 926	1266	1	Ref
	UWL	66 939	506	1.43	(1.27–1.62)
Cancer	No UWL	266 358	4538	1	Ref
	UWL	63 949	1107	1.03	(0.95–1.11)

BMI, body mass index; CHF, congestive heart failure; CI, confidence interval; COPD, chronic obstructive pulmonary disorder; HR, hazard ratio; IBD, inflammatory bowel disease; Ref, reference; UWL, unexpected weight loss.

All models are adjusted for smoking, alcohol consumption, material deprivation, number of comorbidities, and BMI and stratified by matching group (matched on age, sex, and practice).

The appropriateness of the proportionality assumption in the Cox model varied by disease type (*Figure* S1). In *Table* S2, the HRs for different follow‐up intervals (0–2 and 0–6 months) are provided. For many diseases, HRs decreased with longer intervals since the index UWL consultation, reflecting higher excess risk immediately after the index weight loss event. For eating disorders, for example, in the first 2 months after the rUWL, the HR was 37.7, reducing to 12.8 if a 6 month interval was used. The most extreme example was cancer for which the hazards crossed from higher to lower risk in UWL patients relative to comparators around 3 months after diagnosis (*Figure* S1). For dementia, CHF, rheumatoid arthritis, depression, and alcohol addiction, however, HRs remained relatively consistent irrespective of the follow‐up window.

*Post hoc* analyses revealed that most thyroid disorders diagnosed among patients with rUWL were hyperthyroidism (>80%), whereas hypothyroidism was more common in those without (>65%, not shown).

## Discussion

In this study of serious disease among patients with rUWL, we observed that (i) patients with rUWL had significantly higher risk of nearly all serious diseases examined compared with patients without, (ii) the absolute risks of any given serious disease were relatively low (<6% after 1 year), and (iii) the magnitude and rank order of absolute risks varied by age and sex. Depression, diabetes, thyroid disorders, dementia, and cancer were among the most common diagnoses. The highest HRs were for rarer diagnoses such a malabsorption, eating disorders, and IBD.

### Strengths and limitations

This is the largest study to date examining the association between UWL as coded in the medical record and subsequent serious disease diagnosis in primary care. By using a matched cohort design and time‐to‐event methods, we have estimated both absolute risks and HRs. UWL was extracted from primary care records using a carefully defined and internally validated metric as described in a previous publication.^21^ We treated outcomes as independent and avoided informative censoring by allowing patients to remain in the risk set until death, end of follow‐up, or the event of interest. While multiple outcomes were uncommon (only 0.1% of the sample had more than one outcome during the first year of follow‐up), this approach was likely most representative of the real world where the diagnosis of one disease does not preclude an individual from being diagnosed with something else.

This study also has limitations. First, while internally validated, the recording of weight loss in the GP record may capture only a subset of true UWL events, which could lead to measurement bias. This could be overcome through prospective collection of weight measurements within the context of a research study or through analysis of objective weight measurements in healthcare settings where weight is routinely measured. This was not possible using retrospective data from the English NHS where weight recording is not a routine clinical activity.^22^ Second, while we investigated serious diseases reported to be associated with UWL in the literature, other diagnoses not explored in this analysis may occur among patients consulting primary care with UWL, and so our analysis may not represent an exhaustive list of all possible outcomes.

### Comparison with the existing literature

The literature to date has emphasized malignancies, gastrointestinal conditions, and psychiatric conditions as the most likely diagnoses among persons with UWL with probabilities sometimes exceeding 30%.^2^, ^6^, ^10^, ^12^, ^13^, ^14^, ^15^, ^16^, ^17^, ^23^, ^24^ Previous studies have, however, primarily focused on a referred population for whom endocrine disorders may have already been ruled out.^12^, ^13^, ^14^, ^15^, ^17^ Previous studies have also loosely and inconsistently defined UWL. In this cohort of primary care patients, and using an internally validated definition of UWL,^21^ the observed risks were notably lower.

The results presented here illustrate that the follow‐up interval has a significant impact on both the estimates of absolute risk and HRs. However, reviews that have summarized serious disease risk following UWL have amalgamated probabilities from studies with follow‐up ranging from a single admission^25^ to 3 years.^10^ Caution should be exercised when comparing cumulative risks without taking follow‐up time into account. For endocrine disorders that may be more easily diagnosed with a blood test such as diabetes and thyroid disorders, the excess risk was concentrated within the first 2–3 months following the index rUWL consultation in primary care. For diagnoses with potentially more complicated diagnostic workup, the excess risk was sustained for at least a year after diagnosis.

Similar to follow‐up time, few studies have stratified risks by age and sex. The proportion of UWL cohorts made up by men has ranged from 33% to 99%, so when probabilities are not sex stratified, the sex ratio may influence the estimates of cumulative risks.^23^, ^24^ The findings of our analyses suggest that the most likely diagnoses vary significantly according to these factors. For example, cancer is the most common diagnosis in men aged 60–79 with UWL, whereas both depression and thyroid disorders were more likely in women of the same age.

### Implications for research and practice

Our findings have implications for both research and clinical practice. Our findings suggest a range of differential diagnoses associated with UWL, varying in probability by age, sex, and follow‐up time. As most of the studies of UWL have been conducted in secondary care patients, this analysis provides new evidence to patients and clinicians of the risks of serious disease in primary care. Depending on age and sex, the results suggest that once the practitioner observes UWL, workup should include screening for diabetes, thyroid dysfunction, depression, and dementia (*Box* 1). Replicating these analyses in other primary care settings would support the suggestion that, if performed in a timely manner, this workup could be used to triage patients eligible for cancer pathway referral.

**Box 1. Managing patients with unexpected weight loss in primary care**

- In younger men and women (aged <60 years), prioritize screening for depression and testing for thyroid function and diabetes.
- In men aged 60–79, prioritize cancer investigation and testing for diabetes and chronic obstructive pulmonary disorder (especially in smokers).
- In women aged 60–79, prioritize testing for thyroid function and screening for depression while considering cancer investigation.
- In older patients (aged 80 and over), prioritize screening for dementia, depression, and heart failure during workup for cancer investigation.

Clinically, the sustained excess risk observed for diagnoses such as depression and dementia, and relative high probability in certain subgroups (women and older people, respectively) may be suggestive that these diagnoses could be accelerated, which could lead to earlier diagnosis and management if considered at the index consultation for UWL. Alternatively, the apparent delays may signify that these diagnoses are made as a diagnosis of exclusion following testing for diseases that are more easily excluded or considered more serious, such as cancer. When initial investigations are normal, and no immediate diagnosis is made, our results support a safety‐netting strategy of re‐review so that the outstanding possible diagnoses pertinent to the patient's age and sex may be considered at review.

## Funding

B.D.N. was supported by a National Institute for Health and Care Research (NIHR) Doctoral Research Fellowship (DRF‐2015‐08‐18) and Academic Clinical Lectureship. R.H. acknowledges part funding from the NIHR Oxford Medtech and In Vitro Diagnostics Co‐operative and from the NIHR Oxford and Thames Valley Applied Research Collaboration. R.H. and J.O. acknowledge part funding from the NIHR Oxford Biomedical Research Centre. The views expressed are those of the authors and not necessarily those of the NHS, the NIHR, or the Department of Health. The funders had no role in the study design, data collection and analysis, decision to publish, or preparation of the manuscript.

## Conflict of interest

None declared.

## Supporting information


**Table S1.** Cumulative proportion of patients with a serious disease outcome, 0–12 months from index weight loss event.
**Table S2.** Hazard ratios (HRs) and 95% confidence intervals (Cl) for risk of serious disease within twelve months among adults with unexplained weight loss compared to those without.
**Figure S1.** Hazard function for twelve serious diseases after an index unexplained weight loss event (UWL) and in matched comparators. CHF: Coronary Heart Failure; COPD: Chronic Obstructive Pulmonary Disease; IBD: Inflammatory Bowel Disease.
