# Supplementary material for: Serious disease risk among patients with unexpected weight loss: a matched cohort of over 70 000 primary care presentations
Source: J Cachexia Sarcopenia Muscle. 2022 Sep 3;13(6):2661–8. doi: 10.1002/jcsm.13056 (PMC9745555; doi:10.1002/jcsm.13056)
Supplement: Supplementary file 1 — Table S1. Cumulative proportion of patients with a serious disease outcome, 0–12 months from index weight loss event. Table S2. Hazard ratios (HRs) and 95% confidence intervals (Cl) for risk of serious disease within twelve months among adults with unexplained weight loss compared to those without. Figure S1. Hazard function for twelve serious diseases after an index unexplained weight loss event (UWL) and in matched comparators. CHF: Coronary Heart Failure; COPD: Chronic Obstructive Pulmonary Disease; IBD: Inflammatory Bowel Disease. [file JCSM-13-2661-s001.docx]

**SUPPLEMENTARY MATERIALS**

| **Supplementary Table 1.** Cumulative proportion of patients with a serious disease outcome, 0-12 months from index weight loss event. | | | | | | | | | | | | | | | |
| --- | --- | --- | --- | --- | --- | --- | --- | --- | --- | --- | --- | --- | --- | --- | --- |
|  |  |  |  |  |  |  |  |  |  |  |  |  |  |  |  |
|  |  | **Aged<60** | | | |  | **Aged 60-79** | | | |  | **Aged 80+** | | | |
| **Event** |  | **N** | **n** | **%** | **95% CI** |  | **N** | **n** | **%** | **95% CI** |  | **N** | **n** | **%** | **95% CI** |
| **Men** | | | | | | | | | | | | | | | |
| Thyroid disorders | UWL | 13,007 | 187 | 1.44 | (1.25, 1.66) |  | 10,514 | 141 | 1.34 | (1.14, 1.58) |  | 5,066 | 47 | 0.93 | (0.70, 1.23) |
|  | No UWL | 54,537 | 55 | 0.10 | (0.08, 0.13) |  | 45,027 | 120 | 0.27 | (0.22, 0.32) |  | 19,759 | 107 | 0.54 | (0.45, 0.65) |
| Diabetes | UWL | 12,518 | 370 | 2.96 | (2.67, 3.27) |  | 9,139 | 253 | 2.77 | (2.45, 3.13) |  | 4,567 | 53 | 1.16 | (0.86, 1.52) |
|  | No UWL | 51,302 | 191 | 0.37 | (0.32, 0.43) |  | 34,892 | 451 | 1.29 | (1.18, 1.42) |  | 15,979 | 162 | 1.01 | (0.87, 1.18) |
| Depression | UWL | 10,382 | 295 | 2.84 | (2.54, 3.18) |  | 8,881 | 163 | 1.84 | (1.58, 2.14) |  | 4,606 | 107 | 2.32 | (1.93, 2.80) |
|  | No UWL | 39,074 | 253 | 0.65 | (0.57, 0.73) |  | 34,637 | 151 | 0.44 | (0.37, 0.51) |  | 16,983 | 122 | 0.72 | (0.60, 0.86) |
| Cancer | UWL | 12,647 | 53 | 0.42 | (0.31, 0.54) |  | 9,458 | 358 | 3.79 | (3.42, 4.19) |  | 4,340 | 229 | 5.28 | (4.65, 5.98) |
|  | No UWL | 54,636 | 165 | 0.30 | (0.26, 0.35) |  | 41,141 | 1268 | 3.08 | (2.92, 3.25) |  | 17,413 | 791 | 4.54 | (4.24, 4.86) |
| COPD | UWL | 13,013 | 104 | 0.80 | (0.66, 0.97) |  | 9,323 | 234 | 2.51 | (2.21, 2.85) |  | 4,526 | 87 | 1.92 | (1.56, 2.37) |
|  | No UWL | 54,654 | 84 | 0.15 | (0.12, 0.19) |  | 38,290 | 342 | 0.89 | (0.80, 0.99) |  | 16,695 | 174 | 1.04 | (0.90, 1.21) |
| Dementia | UWL | 13,252 | 8 | 0.06 | (0.03, 0.12) |  | 10,707 | 110 | 1.03 | (0.85, 1.24) |  | 5,003 | 144 | 2.88 | (2.45, 3.38) |
|  | No UWL | 56,045 | 4 | 0.01 | (0.00, 0.02) |  | 46,797 | 169 | 0.36 | (0.31, 0.42) |  | 19,547 | 347 | 1.78 | (1.60, 1.97) |
| CHF | UWL | 13,220 | 8 | 0.06 | (0.03, 0.12) |  | 10,312 | 131 | 1.27 | (1.07, 1.51) |  | 4,637 | 123 | 2.65 | (2.23, 3.16) |
|  | No UWL | 55,798 | 26 | 0.05 | (0.03, 0.07) |  | 43,988 | 245 | 0.56 | (0.49, 0.63) |  | 17,133 | 304 | 1.77 | (1.59, 1.98) |
| Eating disorders | UWL | 13,202 | 28 | 0.21 | (0.15, 0.31) |  | 10,930 | 33 | 0.30 | (0.21, 0.42) |  | 5,371 | 19 | 0.35 | (0.23, 0.55) |
|  | No UWL | 55,789 | 4 | 0.01 | (0.00, 0.02) |  | 48,230 | 11 | 0.02 | (0.01, 0.04) |  | 21,973 | 16 | 0.07 | (0.04, 0.12) |
| Rheumatoid arthritis | UWL | 13,098 | 22 | 0.17 | (0.11, 0.25) |  | 10,560 | 81 | 0.77 | (0.62, 0.95) |  | 5,092 | 34 | 0.67 | (0.48, 0.93) |
|  | No UWL | 55,044 | 23 | 0.04 | (0.03, 0.06) |  | 45,267 | 123 | 0.27 | (0.23, 0.32) |  | 19,863 | 53 | 0.27 | (0.20, 0.35) |
| Alcohol addiction | UWL | 12,183 | 92 | 0.76 | (0.62, 0.93) |  | 10,371 | 34 | 0.33 | (0.23, 0.46) |  | 5,339 | 6 | 0.11 | (0.05, 0.25) |
|  | No UWL | 49,913 | 113 | 0.23 | (0.19, 0.27) |  | 44,296 | 66 | 0.15 | (0.12, 0.19) |  | 21,630 | 13 | 0.06 | (0.03, 0.10) |
| IBD | UWL | 13,103 | 48 | 0.37 | (0.28, 0.49) |  | 10,845 | 26 | 0.24 | (0.16, 0.35) |  | 5,369 | 6 | 0.11 | (0.05, 0.25) |
|  | No UWL | 55,094 | 16 | 0.03 | (0.02, 0.05) |  | 47,361 | 16 | 0.03 | (0.02, 0.06) |  | 21,843 | 4 | 0.02 | (0.01, 0.05) |
| Malabsorption | UWL | 13,193 | 46 | 0.35 | (0.26, 0.47) |  | 10,948 | 31 | 0.28 | (0.20, 0.40) |  | 5,410 | 6 | 0.11 | (0.05, 0.25) |
|  | No UWL | 55,680 | 11 | 0.02 | (0.01, 0.04) |  | 48,232 | 10 | 0.02 | (0.01, 0.04) |  | 22,142 | 2 | 0.01 | (0.00, 0.04) |
| **Women** | | | | | | | | | | | | | | | |
| Thyroid disorders | UWL | 17,095 | 400 | 2.34 | (2.12, 2.58) |  | 10,673 | 251 | 2.35 | (2.08, 2.66) |  | 7,757 | 122 | 1.57 | (1.32, 1.88) |
|  | No UWL | 65,902 | 259 | 0.39 | (0.35, 0.44) |  | 40,538 | 307 | 0.76 | (0.68, 0.85) |  | 27,591 | 233 | 0.84 | (0.74, 0.96) |
| Diabetes | UWL | 17,787 | 119 | 0.67 | (0.56, 0.80) |  | 11,095 | 169 | 1.52 | (1.31, 1.77) |  | 8,279 | 72 | 0.87 | (0.69, 1.09) |
|  | No UWL | 70,191 | 175 | 0.25 | (0.22, 0.29) |  | 43,191 | 397 | 0.92 | (0.83, 1.01) |  | 30,722 | 236 | 0.77 | (0.68, 0.87) |
| Depression | UWL | 12,096 | 452 | 3.74 | (3.41, 4.09) |  | 8,612 | 212 | 2.46 | (2.15, 2.81) |  | 6,892 | 159 | 2.31 | (1.98, 2.69) |
|  | No UWL | 39,090 | 478 | 1.22 | (1.12, 1.34) |  | 29,488 | 234 | 0.79 | (0.70, 0.90) |  | 23,263 | 218 | 0.94 | (0.82, 1.07) |
| Cancer | UWL | 17,863 | 37 | 0.21 | (0.15, 0.29) |  | 11,112 | 239 | 2.15 | (1.9, 2.44) |  | 8,229 | 191 | 2.32 | (2.02, 2.67) |
|  | No UWL | 71,740 | 335 | 0.47 | (0.42, 0.52) |  | 47,621 | 1070 | 2.25 | (2.12, 2.38) |  | 33,807 | 909 | 2.69 | (2.52, 2.87) |
| COPD | UWL | 17,997 | 105 | 0.58 | (0.48, 0.71) |  | 11,139 | 218 | 1.96 | (1.72, 2.23) |  | 8,511 | 81 | 0.95 | (0.77, 1.18) |
|  | No UWL | 72,133 | 100 | 0.14 | (0.11, 0.17) |  | 45,702 | 350 | 0.77 | (0.69, 0.85) |  | 33,265 | 223 | 0.67 | (0.59, 0.76) |
| Dementia | UWL | 18,347 | 7 | 0.04 | (0.02, 0.08) |  | 12,212 | 186 | 1.52 | (1.32, 1.76) |  | 8,133 | 297 | 3.65 | (3.27, 4.08) |
|  | No UWL | 73,998 | 5 | 0.01 | (0.00, 0.02) |  | 52,232 | 224 | 0.43 | (0.38, 0.49) |  | 31,427 | 690 | 2.20 | (2.04, 2.36) |
| CHF | UWL | 18,333 | 8 | 0.04 | (0.02, 0.09) |  | 12,186 | 89 | 0.73 | (0.59, 0.90) |  | 8,254 | 147 | 1.78 | (1.52, 2.09) |
|  | No UWL | 73,891 | 12 | 0.02 | (0.01, 0.03) |  | 51,705 | 221 | 0.43 | (0.37, 0.49) |  | 31,411 | 458 | 1.46 | (1.33, 1.60) |
| Eating disorders | UWL | 17,911 | 102 | 0.57 | (0.47, 0.69) |  | 12,501 | 47 | 0.38 | (0.28, 0.50) |  | 9,248 | 66 | 0.71 | (0.56, 0.91) |
|  | No UWL | 71,586 | 30 | 0.04 | (0.03, 0.06) |  | 54,081 | 18 | 0.03 | (0.02, 0.05) |  | 38,425 | 27 | 0.07 | (0.05, 0.10) |
| Rheumatoid arthritis | UWL | 18,027 | 39 | 0.22 | (0.16, 0.30) |  | 11,643 | 122 | 1.05 | (0.88, 1.25) |  | 8,502 | 45 | 0.53 | (0.40, 0.71) |
|  | No UWL | 71,870 | 79 | 0.11 | (0.09, 0.14) |  | 47,723 | 206 | 0.43 | (0.38, 0.49) |  | 32,578 | 143 | 0.44 | (0.37, 0.52) |
| Alcohol addiction | UWL | 17,827 | 57 | 0.32 | (0.25, 0.41) |  | 12,362 | 24 | 0.19 | (0.13, 0.29) |  | 9,328 | 5 | 0.05 | (0.02, 0.13) |
|  | No UWL | 71,054 | 69 | 0.10 | (0.08, 0.12) |  | 53,035 | 32 | 0.06 | (0.04, 0.09) |  | 38,808 | 4 | 0.01 | (0.00, 0.03) |
| IBD | UWL | 18,150 | 50 | 0.28 | (0.21, 0.36) |  | 12,472 | 25 | 0.20 | (0.14, 0.30) |  | 9,306 | 12 | 0.13 | (0.07, 0.23) |
|  | No UWL | 72,708 | 29 | 0.04 | (0.03, 0.06) |  | 53,510 | 21 | 0.04 | (0.03, 0.06) |  | 38,557 | 10 | 0.03 | (0.01, 0.05) |
| Malabsorption | UWL | 18,254 | 57 | 0.31 | (0.24, 0.40) |  | 12,555 | 34 | 0.27 | (0.19, 0.38) |  | 9,346 | 5 | 0.05 | (0.02, 0.13) |
|  | No UWL | 73,372 | 27 | 0.04 | (0.03, 0.05) |  | 54,191 | 16 | 0.03 | (0.02, 0.01) |  | 38,948 | 5 | 0.01 | (0.01, 0.03) |

CI: Confidence interval; UWL: Unexpected weight loss; CHF: Coronary Heart Failure; COPD: Chronic Obstructive Pulmonary Disease; IBD: Inflammatory Bowel Disease.

**Supplementary Figure 1.** Hazard function for twelve serious diseases after an index unexplained weight loss event (UWL) and in matched comparators. CHF: Coronary Heart Failure; COPD: Chronic Obstructive Pulmonary Disease; IBD: Inflammatory Bowel Disease.
